# Post-Traumatic Chest Wall Abscess Associated with *Gordonia sputi* in an Immunocompetent Patient: A Case Report

**DOI:** 10.70352/scrj.cr.26-0054

**Published:** 2026-06-11

**Authors:** Momoko Soda, Hideki Motoyama, Taishi Adachi, Takao Nakanishi, Takuya Ikenari, Seiko Nasu, Mitsugu Omasa

**Affiliations:** 1 Department of Thoracic Surgery, Kobe City Nishi-Kobe Medical Center, Kobe, Hyogo, Japan; 2 Department of Clinical Laboratory, Kobe City Nishi-Kobe Medical Center, Kobe, Hyogo, Japan; 3 Department of Clinical Laboratory, Kobe City Medical Center General Hospital, Kobe, Hyogo, Japan

**Keywords:** chest wall abscess, *Gordonia sputi*, post-traumatic infection, immunocompetent host

## Abstract

**INTRODUCTION:**

*Gordonia* species are rare aerobic Gram-positive actinomycetes and opportunistic pathogens. Their clinical manifestations and optimal management remain poorly defined. *Gordonia sputi* is an especially uncommon cause of soft-tissue and chest wall infections, and diagnosis is often delayed because of slow growth and difficulties in identification.

**CASE PRESENTATION:**

We report the case of a 45-year-old previously healthy man who presented with a progressively enlarging and painful mass on the left chest wall following blunt trauma. He was afebrile and had no systemic symptoms. Contrast-enhanced CT revealed a localized chest wall abscess, and surgical drainage was performed. Initial cultures from the 1st debridement were negative, but histopathological examination showed granulomatous inflammation with features suggestive of infection by an actinomycete-like organism. Because of wound dehiscence, a 2nd debridement was required. Microbiological examination of specimens obtained during the second procedure was positive on Kinyoun staining. Matrix-assisted laser desorption/ionization time-of-flight mass spectrometry (MALDI-TOF MS) suggested *G. sputi*, and this identification was confirmed by 16S ribosomal RNA gene sequencing. Based on antimicrobial susceptibility testing, oral amoxicillin was administered for 6 months. The wound healed completely, and no recurrence was observed during 1 year of follow-up.

**CONCLUSIONS:**

This case highlights the diagnostic challenges associated with *G. sputi* isolated from a post-traumatic chest wall abscess in an immunocompetent patient. Accurate diagnosis required a combination of histopathology, MALDI-TOF MS, and molecular identification. In addition, successful management required both adequate surgical drainage and prolonged, pathogen-directed antimicrobial therapy. Because the organism was isolated only after re-debridement and the initial culture was negative, the precise timing and causal role of *G. sputi* in the infection cannot be determined conclusively.

## INTRODUCTION

*Gordonia* species are aerobic, Gram-positive actinomycetes belonging to the order Actinomycetales. Phylogenetically, they are related to *Rhodococcus*, *Tsukamurella*, and fast-growing nontuberculous mycobacteria.^1,2)^ They are widely distributed in soil, water, and other environmental sources and were traditionally considered nonpathogenic microorganisms.^1)^ However, in recent years, reports of human infections caused by *Gordonia* species have increased, particularly in immunocompromised patients and those with indwelling medical devices.^2,3)^ Reported clinical manifestations include bloodstream infections, brain abscesses, and catheter-associated infections.^2–4)^

Despite increasing case reports, diagnosis remains challenging. Bacteria of the genus *Gordonia* grow slowly in routine bacterial cultures and are frequently misidentified by conventional biochemical methods as *Corynebacterium*, *Rhodococcus*, or fast-growing *Mycobacterium* species.^3,4)^ Advances in microbial diagnostic technologies, including matrix-assisted laser desorption/ionization time-of-flight mass spectrometry (MALDI-TOF MS) and 16S ribosomal RNA (rRNA) gene sequencing, have significantly improved species-level identification accuracy.^3,5)^

Optimal treatment strategies remain unestablished, and antimicrobial susceptibility patterns vary among isolates.^2)^ Past reports suggest that achieving complete cure, particularly in deep-seated and healthcare-associated infections, may require prolonged antimicrobial therapy (sometimes combined with surgical intervention).^2,4)^

Given the rarity of *Gordonia sputi* infections and the lack of standardized management guidelines, further case reports are essential for improving clinical recognition and establishing evidence-based treatment strategies.

## CASE PRESENTATION

A 45-year-old man with no significant past medical history presented with a painful mass on the left chest wall. Approximately 3 months prior to presentation, he sustained a blunt trauma to his left chest wall during on-site work but did not seek immediate medical attention. The patient was initially evaluated at another hospital; however, no detailed documentation of the skin condition at the time of injury was available. According to the patient, there were no obvious open wounds, such as abrasions or lacerations. Persistent pain gradually limited his activities, prompting him to later visit a local clinic. He was diagnosed with an incomplete fracture of the left ribs and prescribed pain medication. Two months after the injury, erythema and swelling developed at the same site. MRI revealed hematoma formation, and secondary infection was suspected, leading to referral to our hospital (Kobe City Nishi-Kobe Medical Center).

At the initial visit, the patient was afebrile with no systemic fever. He measured 158.5 cm in height and weighed 43.7 kg. Physical examination revealed a warm, erythematous, tumor-like lesion extending dorsally along the left chest wall; no skin fistula formation was observed. Laboratory findings showed an elevated white blood cell count (10700/μL; neutrophils 83.3%) and an elevated C-reactive protein (18.1 mg/dL). Hemoglobin was 11.0 g/dL, and liver and kidney function values were within normal ranges. HbA1c was 5.9%. Contrast-enhanced CT revealed a 10 × 5-cm hypodense mass located extrapleurally on the left chest wall at the level of the 7th to 10th ribs, with no internal enhancement (**Fig. 1**). Segmental atelectasis of the left lower lobe was noted, but no pleural effusion was present.

**Fig. 1:**
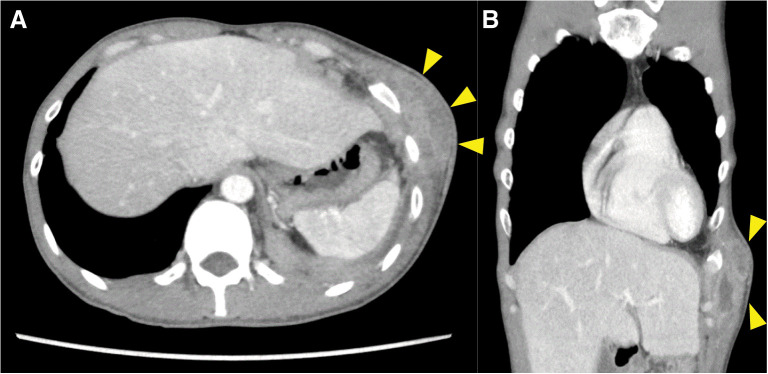
Contrast-enhanced chest CT from a case of chest wall abscess associated with *Gordonia sputi* in an immunocompetent individual. The abscess is seen in the left chest wall on axial (**A**) and coronal (**B**) images (arrowheads).

Needle aspiration was attempted in the outpatient setting, but no specimen was obtained. Oral levofloxacin (500 mg/day) was initiated. Five days later, the patient was admitted for surgical treatment, and intravenous ampicillin/sulbactam (3 g every 6 h) was started.

The day after admission, under general anesthesia, an abscess excision and extensive debridement of devitalized subcutaneous and muscle tissue were performed. The intercostal lesion was carefully dissected to avoid pleural injury. A drain was placed, and the wound was primarily sutured. The drain was removed on POD 2, and the patient was discharged on POD 4 with oral amoxicillin (500 mg)–clavulanate (125 mg), 3 times daily.

Histopathological examination was positive for acute inflammation, abscess formation, hemorrhage, and hematoma. Morphologically, filamentous bacterial colonies consistent with actinomycete-like microorganisms were also observed (**Fig. 2**). No microorganisms were isolated from the intraoperative culture. Sutures were removed 1 week after discharge; however, wound dehiscence occurred 1 week later, requiring repeat surgical debridement. Histopathological examination of the 2nd debridement specimen again demonstrated filamentous bacterial colonies consistent with an actinomycete-like organism.

**Fig. 2:**
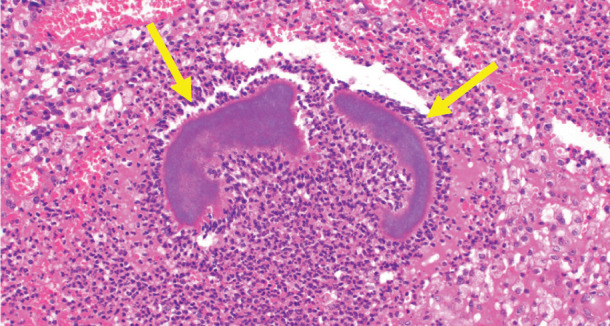
Hematoxylin and eosin staining revealed active inflammation with abundant neutrophilic infiltration, abscess formation, and hemorrhage. Bacterial colonies morphologically consistent with actinomycete-like microorganisms were also identified (arrows).

Specimens obtained during the procedure were submitted for microbiological examination. Two days after inoculation onto TSA II medium (5% sheep blood agar), pale yellow colonies resembling *Corynebacterium*-like organisms were observed. Kinyoun staining demonstrated weakly acid-fast bacilli, raising suspicion of *Gordonia* or *Rhodococcus* species (**Fig. 3**). Following subculture, repeated MALDI-TOF MS identified the organism as *G. sputi*. Antimicrobial susceptibility testing showed low MIC values across multiple antibiotics. However, biochemical characterization using the API Coryne system (bioMérieux SA, Marcy-l’Étoile, France) yielded inconclusive results. Definitive identification was subsequently achieved by 16S rRNA gene sequencing, which demonstrated 100% identity with reference strains of *G. sputi*. Based on these findings, antimicrobial therapy was changed to oral amoxicillin (500 mg) 3 times daily for 6 months. At the 1-year follow-up, the patient had no recurrence, and the wound had completely healed.

**Fig. 3:**
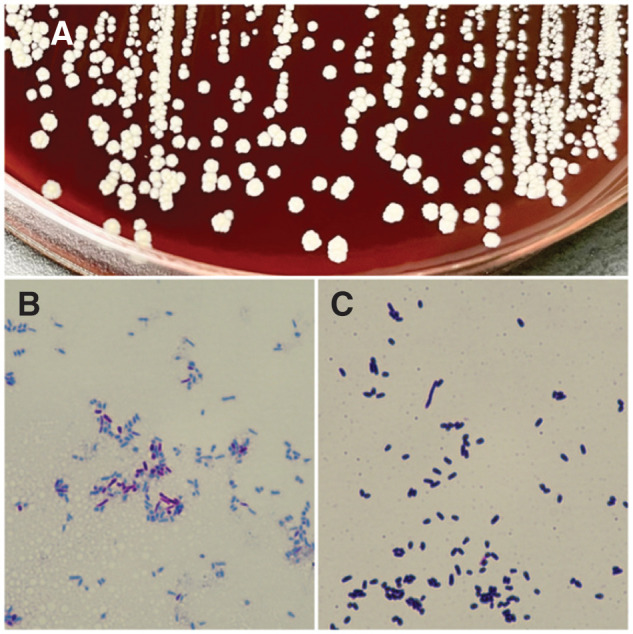
Biochemical confirmation of the pathogen from a case of chest wall abscess associated with *Gordonia sputi* in an immunocompetent individual. (**A**) Colony morphology appeared rough and pale yellow on the surface. (**B**) Kinyoun staining showed weakly acid-fast, light-red organisms. (**C**) Gram-positive rods were observed.

## DISCUSSION

We report an extremely rare case of post-traumatic chest wall abscess associated with *G. sputi* in a healthy male in his 40s. Its ubiquitous presence in the environment may explain the occurrence of infection even in otherwise healthy individuals without a clear exposure history. *Gordonia* was historically classified within *Rhodococcus*, but advances in 16S rRNA gene sequence analysis in the late 1980s led to a taxonomic reevaluation, establishing it as an independent genus. Of the 13 known species, 5, including *G. sputi*, have been reported as opportunistic human pathogens.^6)^

Human infections caused by *Gordonia* remain rare. Reported cases include catheter-associated bloodstream infections and infections in immunocompromised hosts,^7)^ such as solid organ transplant recipients and patients with hematologic malignancies. Localized soft-tissue infections in immunocompetent individuals are particularly uncommon. Past reports have documented brain abscesses caused by *G. sputi* in human immunodeficiency virus–infected patients.^8)^ However, to our knowledge, no previous literature has identified a chest wall abscess associated with *Gordonia* species. Therefore, this case represents the first reported instance of a traumatic chest wall infection in an immunocompetent host, expanding the known clinical spectrum of *Gordonia* infections.

One factor contributing to the low number of reported cases is the inherent difficulty in microbiological identification. On Gram staining, *Gordonia* species may appear as elongated Gram-positive rods resembling *Corynebacterium*, leading to misclassification or delayed recognition. In addition, these organisms may exhibit weak acid-fastness and relatively slow growth, further complicating routine laboratory identification. Although MALDI-TOF MS has improved the identification of uncommon pathogens, conventional biochemical identification systems may still yield inconclusive results. Therefore, molecular methods such as 16S rRNA gene sequencing may be required for definitive identification when rare actinomycete-like organisms are suspected.

In the present case, definitive identification of *G. sputi* was achieved only after multiple microbiological techniques were applied, including staining, MALDI-TOF MS, and ultimately 16S rRNA gene sequencing. This case highlights the importance of considering uncommon actinomycete-like organisms and using advanced diagnostic methods when routine identification remains inconclusive.

In the present case, intraoperative cultures obtained during the 1st debridement were negative. However, histopathological examination of the resected abscess specimen demonstrated acute inflammation, abscess formation, hemorrhage, hematoma, and filamentous bacterial colonies consistent with an actinomycete-like organism. This finding indicates that an infectious process was already present in the initial lesion, even though the causative organism could not be microbiologically identified at that time.

In addition, histopathological examination of the 2nd debridement specimen again revealed filamentous bacterial colonies with similar morphology. This concordance between the 1st and 2nd specimens supports the presence of an actinomycete-like infection within the lesion from the outset, rather than a purely secondary colonization or contamination. The subsequent identification of *G. sputi* in the 2nd specimen therefore suggests that this organism was likely involved in the initial infectious process, although the exact timing cannot be determined conclusively from the available data.

*Gordonia* species are generally slow-growing organisms, often requiring 48–72 h of incubation on blood agar before adequate colony formation is observed. This slow growth may contribute to delayed detection in clinical specimens, particularly when cultures are examined only after the standard 24-h incubation period.^9)^ In this context, the negative initial culture may reflect limitations of microbiological detection, including low bacterial burden, sampling variability, prior antimicrobial exposure, or the delayed growth characteristics of *Gordonia* species.

Taken together, this case highlights the diagnostic challenges associated with rare actinomycete-like pathogens in post-traumatic chest wall abscesses and underscores the importance of combining histopathological evaluation with advanced microbiological methods, including MALDI-TOF MS and molecular identification, for accurate diagnosis and management.

The optimal antimicrobial therapy for *G. sputi* remains unestablished due to the limited number of cases and the absence of standardized susceptibility testing guidelines. Past reports have shown variability in susceptibility patterns, with inconsistent reports of susceptibility to β-lactams, tetracyclines, carbapenems, aminoglycosides, and macrolides. Based on the susceptibility data from this case, therapy was switched to oral amoxicillin. This decision was partially guided by the treatment principles for *Actinomyces* species (where long-term antibiotic therapy exceeding 6 months is recommended).^10)^ The clinical improvement observed after 1 year and the absence of recurrence suggest that long-term monotherapy with amoxicillin, based on susceptibility testing, may be effective.

Another notable feature of this case is its possible association with blunt chest trauma.^11)^ Although abscess formation after trauma is uncommon, it may occur when a hematoma becomes secondarily infected. From a surgical perspective, devitalized tissue, hematoma formation, and impaired local perfusion after trauma can create a microenvironment that predisposes even immunocompetent patients to infection. Hematomas in particular may serve as a nidus for bacterial colonization because of their relatively avascular nature, which limits host immune responses and antibiotic penetration. In the present case, the patient sustained a closed chest injury followed by suspected hematoma formation, persistent inflammation, and delayed erythema and swelling. Imaging studies revealed atelectasis in the left lower lung field; however, intraoperative findings showed no communication between the abscess and the pleural cavity, making a pulmonary origin or empyema unlikely.

Furthermore, repeat surgical debridement played a critical role not only in achieving adequate source control but also in obtaining appropriate specimens for microbiological analysis, which ultimately enabled the definitive identification of *G. sputi*. This highlights the importance of aggressive surgical management and adequate sampling in cases of persistent or atypical soft-tissue infection.

This case highlights several important clinical considerations. First, when acid-fast, slow-growing Gram-positive bacteria are identified, particularly if initial cultures are inconclusive or soft-tissue infections fail to respond to standard therapy, *Gordonia* infection should be included in the differential diagnosis. Second, accurate microbiological diagnosis may require repeated specimen collection, prolonged culture periods, and nucleic acid-based molecular diagnostics. Third, treatment requires a multidisciplinary approach incorporating prolonged antimicrobial therapy based on species identification and susceptibility testing, in addition to surgical drainage or debridement.

## CONCLUSIONS

In conclusion, we report the first known case of post-traumatic chest wall abscess associated with *G. sputi* in an immunocompetent patient. This case highlights the diagnostic difficulty of this rare pathogen and demonstrates that mass spectrometry was essential for its identification. Establishing standardized antimicrobial regimens and optimal treatment durations requires further accumulation of cases and collection of long-term clinical data.

## DECLARATIONS

### Availability of data and materials

All data generated or analyzed during this study are included in this published article.
